# miR-424-5p regulates apoptosis and cell proliferation via targeting Bcl2 in nucleus pulposus cells

**DOI:** 10.1080/19768354.2020.1775699

**Published:** 2020-06-23

**Authors:** Hua-tuo Lu, Yong-qing Xu, Hai Wang, Xu-lin Zhang

**Affiliations:** a Graduate school of kunming medical university, Kunming, PR People’s Republic of China; b Department of orthopedics, 920th Hospital of Joint Logistics Support Force, Kunming, PR People’s Republic of China; c Kunming university of science and technology, Kunming, PR People’s Republic of China; d Department of orthopedics, xingsha branch of hunan provincianal people’s hospital, Changsha, PR People’s Republic of China.

**Keywords:** Intervertebral disc degeneration, mir-424-5p, Bcl2, apoptosis

## Abstract

miRNAs play an important role in the pathogenesis of intervertebral disc degeneration (IDD). The role and the underlying mechanism of miR-424-5p in human nucleus pulposus (NP) are still unknown. We aimed to explore the role of miR-424-5p in IDD.

Real-time PCR was used to detect the expression of miR-424-5p and Bcl2 in IDD tissues and idiopathic scoliosis tissues. Human NP cells were used in our study. MTT and Hoechst apoptosis assays were used to detect the proliferation and apoptosis of NP cells, respectively. Western blotting assays were used to detect the expression levels of Bcl-2, cleaved caspase-3, cleaved caspase-9, caspase-3 and caspase-9 in degenerative NP cells. A luciferase reporter assay was applied to confirm the relationship between miR-424-5p and Bcl2.

Our results showed that the expression of miR-424-5p was increased and Bcl2 was decreased in degenerative NP cells. miR-425-5p expression was negatively correlated with Bcl2 expression in IDD tissues. Suppression of miR-424-5p using an inhibitor increased Bcl2 expression at both the mRNA and protein levels, and it promoted cell viability and inhibited apoptosis. Furthermore, the levels of cleaved caspase-3 and cleaved caspase-9 were downregulated in miR-424-5p-silenced NP cells. Interestingly, we found that silencing miR-424-5p increased p62 expression at both the mRNA and protein levels. Finally, a luciferase reporter assay verified the binding of the miR-424-5p and the 3’UTR of Bcl2.

These results suggested that silencing miR-424-5p suppressed NP cell apoptosis by upregulating Bcl2. Therefore, miR-424-5p might be a novel target for IDD therapies.

## Introduction

Intervertebral disc degeneration (IDD) is a common degenerative spinal disease that leads to intervertebral disc herniation, musculoskeletal disability, spinal canal stenosis, lower back pain and poor quality of life (Sudo et al. 2013; Liu et al. 2015; Wang et al. 2015). Genetic and environmental factors are risk factors for IDD, and genetic factors are the main cause of IDD, accounting for 70% of cases (Hangai et al. 2008; Battie et al. 2009). However, the molecular mechanism behind IDD is still largely unknown.

miRNAs (microRNAs) are one type of noncoding RNA with a length of 20–23 nt that play functional roles by directly binding to the 3’UTR (untranslated region) of target genes. miRNAs are involved in the progression of IDD. miR-143-5p is upregulated in intervertebral discs (IVDs) and promotes the progression of IDD by targeting and regulating eEF2 (Yang et al. 2019). miR-222-3p is also highly expressed in IDD, and enhanced expression of miR-222-3p significantly promotes apoptosis and inhibits the proliferation of nucleus pulposus (NP) cells. Mechanistically, miR-222-3p promotes MMP3 secretion and reduces aggrecan and collagen type II production by directly targeting cyclin-dependent kinase inhibitor 1B (CDKN1B) (Liu et al. 2019). miR-625-5p is upregulated in IDD and lipopolysaccharide (LPS)-treated human NP cells and human annulus fibrosus cells (hAFCs), and it targets collagen type I alpha 1 (COL1A1) (Shen et al. 2019). miR-194-5p is downregulated in IDD tissues, and downregulation of miR-194-5p promotes the progression of IDD by negatively regulating the target genes CUL4A and CUL4B (Chen et al. 2019). Importantly, our study found that miR-424-5p was overexpressed in IDD samples and that it regulated the apoptosis and viability of NP cells.

In the present study, we explored the roles and underlying mechanisms of miR-424-5p in IDD progression. To the best of our knowledge, our findings suggest a novel candidate therapeutic target for IDD therapy.

## Methods

### Clinical samples

Twenty IDD tissues and twenty idiopathic scoliosis tissues were collected from the Department of Orthopaedics, 920th Hospital of Joint Logistics Support Force, Kunming, Yunnan Province, PR China. All study procedures were approved by the Research Ethics Committee of Kunming University of Science and Technology. All samples were collected and stored at −80 °C, and we obtained informed consent from all patients.

### Cell culture

Human primary nucleus pulposus cells were isolated according to a previous study (Wang et al. 2018). Degenerated NP tissues were first diced into small fragments, then they were incubated with 0.25% trypsin solution for 25 min, and then cultured at 37 °C in DMEM (Gibco, USA) containing 10% FBS, 100 U/ml penicillin and 100 μg/ml streptomycin (Gibco, USA) in an atmosphere of 5% CO_2_. First- and second-generation cells were used in our study.

### Cell transfection

NP cells were seeded into 6-well plates (1 × 10^6^ cells/well). NP cells were transfected with an inhibitor control (miR-con) or a miR-424-5p inhibitor (GenePharma Co., Ltd., Shanghai, China) at a concentration of 50 nM using Lipofectamine 3000 (Invitrogen, USA) according to the manufacturer’s instructions. Transfection efficiency was verified by real-time PCR.

### Real-time PCR

TRIzol Reagent (Invitrogen, USA) was used to isolate total RNA according to the manufacturer’s protocol. The QuantiTect reverse transcription kit (Qiagen) was used to synthesize cDNA. The Hairpin-itTM miR-424-5p qRT-PCR Primer Set (GenePharma Co., Ltd., Shanghai, China) was used to detect the expression of miR-424-5p, and U6 was the reference gene. Bcl2 was detected by using SYBR green qRT-PCR assay (Takara), and GAPDH was the endogenous control. The primers were as follows: Bcl2 forward, 5′-GGTGGGGTCATGTGTGTGG-3′ and reverse, 5′-CGGTTCAGGTACTCAGTCATCC-3′; U6 forward, 5′- CCCCTGGATCTTATCAGGCTC-3′ and reverse, 5′- GCCATCTCCCCGGACAAAG-3′; and GAPDH forward, 5′- GGAG CGAGATCCCTCCAAAAT-3′ and reverse, 5′- GGCTGTTGTCATACTTCTCATGG-3′. Quantitative measurements were evaluated using the 2-ΔΔCt method.

### MTT assay

Cell viability was analysed by the MTT assay. After transfection for 24 h, 5 × 10^3^ cells/well were seeded into 96-well plates and were cultured for 48 h. Then, 20 μl of MTT reagent was added to each well. After incubation for 4 h at 37 °C, 200 μl of DMSO was added to each well. The absorbance was detected at 490 nm on a plate reader (Bio-Rad 680).

### Western blot analysis

NP cells were collected and lysed in RIPA lysis buffer (Beyotime Biotechnology, China) for 25 min on ice. The protein concentration was determined using a BCA protein assay. Twelve percent SDS-PAGE gels were used to separate equal amounts of proteins, which were then transferred to PVDF membranes. Then, the membranes were blocked with 5% nonfat milk for 1 h. The membranes were treated with primary antibodies overnight at 4 °C. Next, the membranes were treated with secondary antibodies for 1 h at room temperature. The protein signals were detected by ECL Detection Reagent (Solarbio, Beijing, China). Anti-Bcl2 (1:1000; cat. no. 15071), anti-cleaved caspase-3 (1:1000; cat. no. 9664), anti-cleaved caspase-9 (1:1000; cat. no. 9505), anti-caspase-3 (1:1000; cat. no. 9662), anti-caspase-9 (1:1000; cat. no. 9502), anti-p62 (1:1000; cat. no. 88588) and anti-GAPDH (1:5000; cat. no. 5174) antibodies were purchased from Cell Signaling Technology (Boston, MA, USA). GAPDH was used as an internal control.

### Hoechst staining

NP cells were seeded onto 12-well plates. After transfection for 48 h, the cells were incubated with Hoechst 33342 (Sigma-Aldrich) for 1 h at room temperature. After washing with PBS, the results were examined. Darkly stained condensed nuclei indicated apoptotic cells.

### Luciferase reporter assay

Constructs with wild type (WT) or mutant (MUT) miR-424-5p binding sites were established and were named Bcl2 3′-UTR-Luc vectors. NP cells were seeded into 96-well plates. Twenty-four hours later, vectors were cotransfected with miR-424-5p mimics or a mimic control using Lipofectamine 3000. After incubating for 48 h, NP cells were retrieved and analysed using a luciferase reporter assay system.

### Statistical analysis

Data are presented as the mean ± standard deviation. All experiments were performed three times. Statistical analysis was performed using SPSS 19.0 software (SPSS, Chicago, IL, USA). Data were analysed using Student’s t test (two groups) or analysis of variance (ANOVA, more than two groups). *p* < 0.05 was defined as a statistically significant difference.

## Results

### Expression of mir-424-5p and Bcl2 in patients with IDD

**Figure 1.**
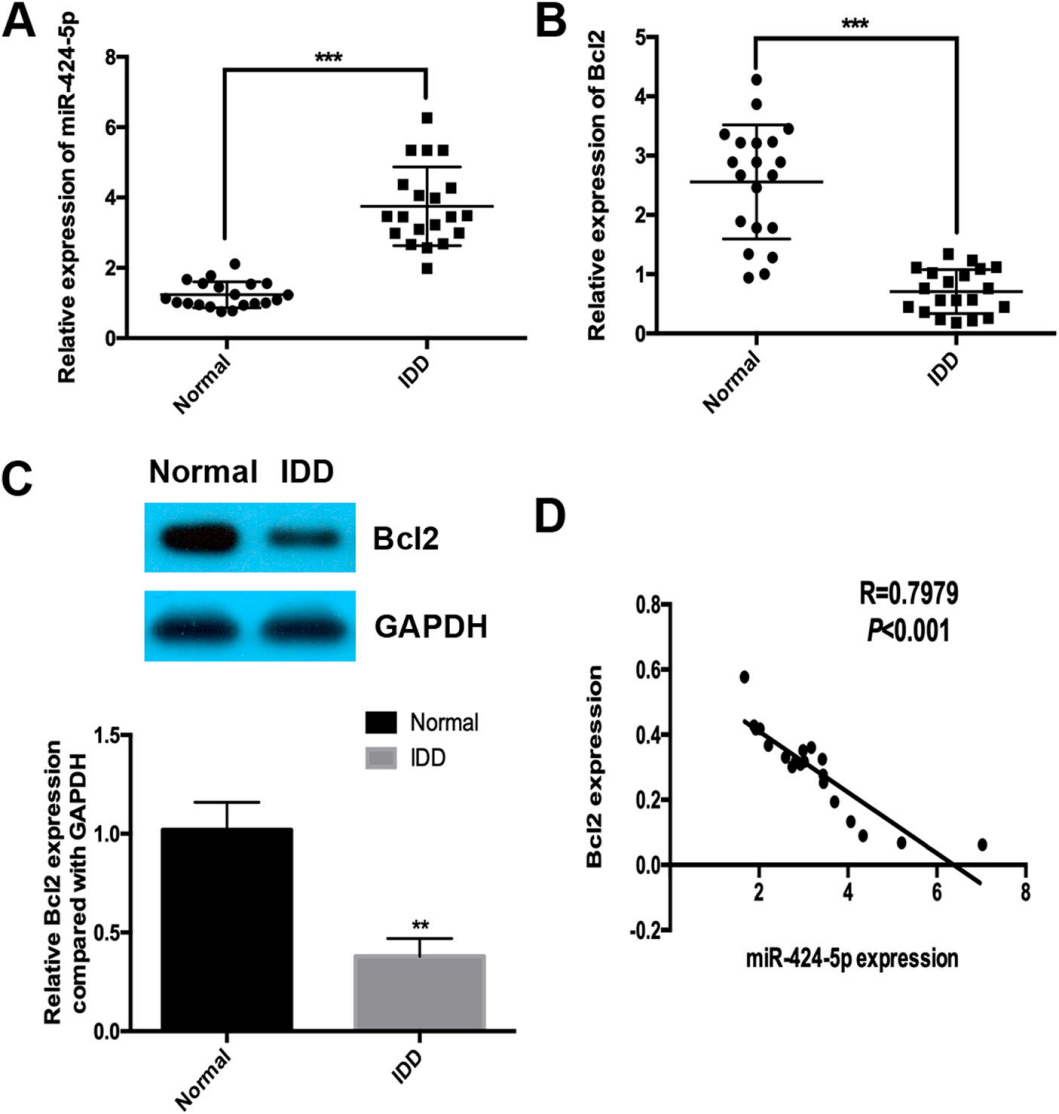
Expression of miR-424-5p and Bcl2 in patients with IDD. (A and B) Real-time PCR was used to detect the expression of miR-424-5p and Bcl2 in IDD tissues (n=20) and normal tissues (idiopathic scoliosis tissues, n=20). (C) Western blotting was used to detect the protein expression of Bcl2 in IDD and normal tissues. (D) Correlation analysis of miR-424-5p expression and Bcl2 mRNA expression in IDD tissues. **, *p* < 0.01; ***, *p* < 0.001.

To explore the role and mechanism of miR-424-5p and its predicted target Bcl2 in IDD, we first analysed the expression levels of these two molecules using real-time PCR in IDD and normal tissue (idiopathic scoliosis tissues). The results showed that miR-424-5p was significantly upregulated in IDD tissues and that Bcl2 was downregulated in IDD tissues compared with the normal group (Figure 1A and B). Western blotting assays showed that Bcl2 was also downregulated in IDD tissues at the protein expression level (Figure 1C). We also found that the mRNA expression level of miR-424-5p was negatively correlated with the mRNA levels of Bcl2 in IDD tissues (Figure 1D, R=0.7979).

### Inhibition of mir-424-5p increases Bcl2 expression in degenerative NP cells

**Figure 2.**
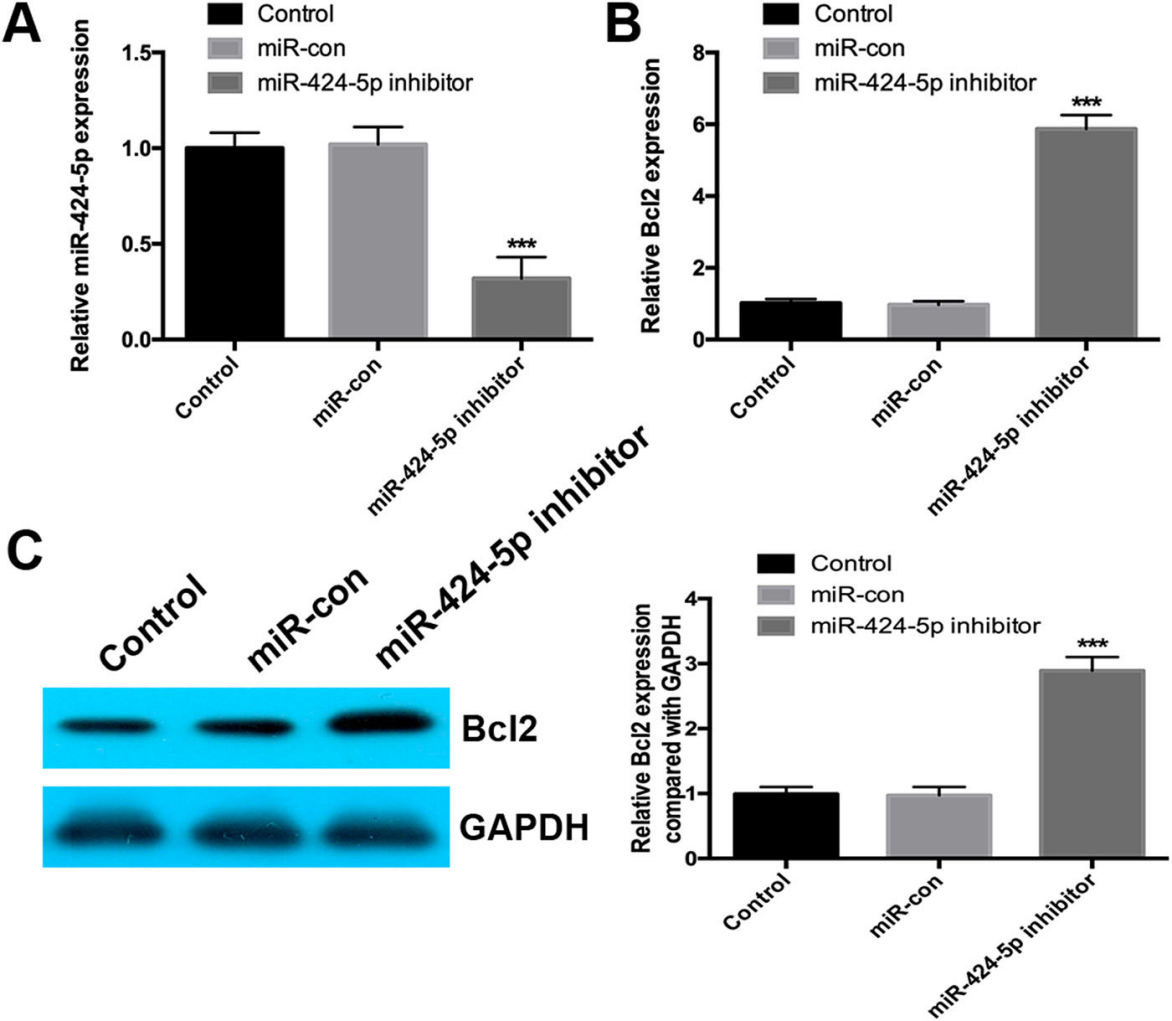
Inhibition of miR-424-5p increases Bcl2 expression in degenerative NP cells. (A and B) Real-time PCR was used to detect the expression of miR-424-5p and Bcl2 in NP cells. (C) Western blotting was used to detect the protein expression of Bcl2 in NP cells. Experiments were performed three times. ***, *p* < 0.001.

We silenced the expression of miR-424-5p using an inhibitor in degenerative NP cells, and the efficiency of miR-424-5p silencing was verified by real-time PCR (Figure 2A). As shown in Figure 2B, inhibition of miR-424-5p significantly increased the Bcl2 mRNA and protein levels (Figure 2B and C). The above results suggested that miR-424-5p negatively regulated Bcl2 in degenerative NP cells.

### Inhibition of mir-424-5p promotes cell viability and suppresses cell apoptosis in degenerative NP cells

**Figure 3.**
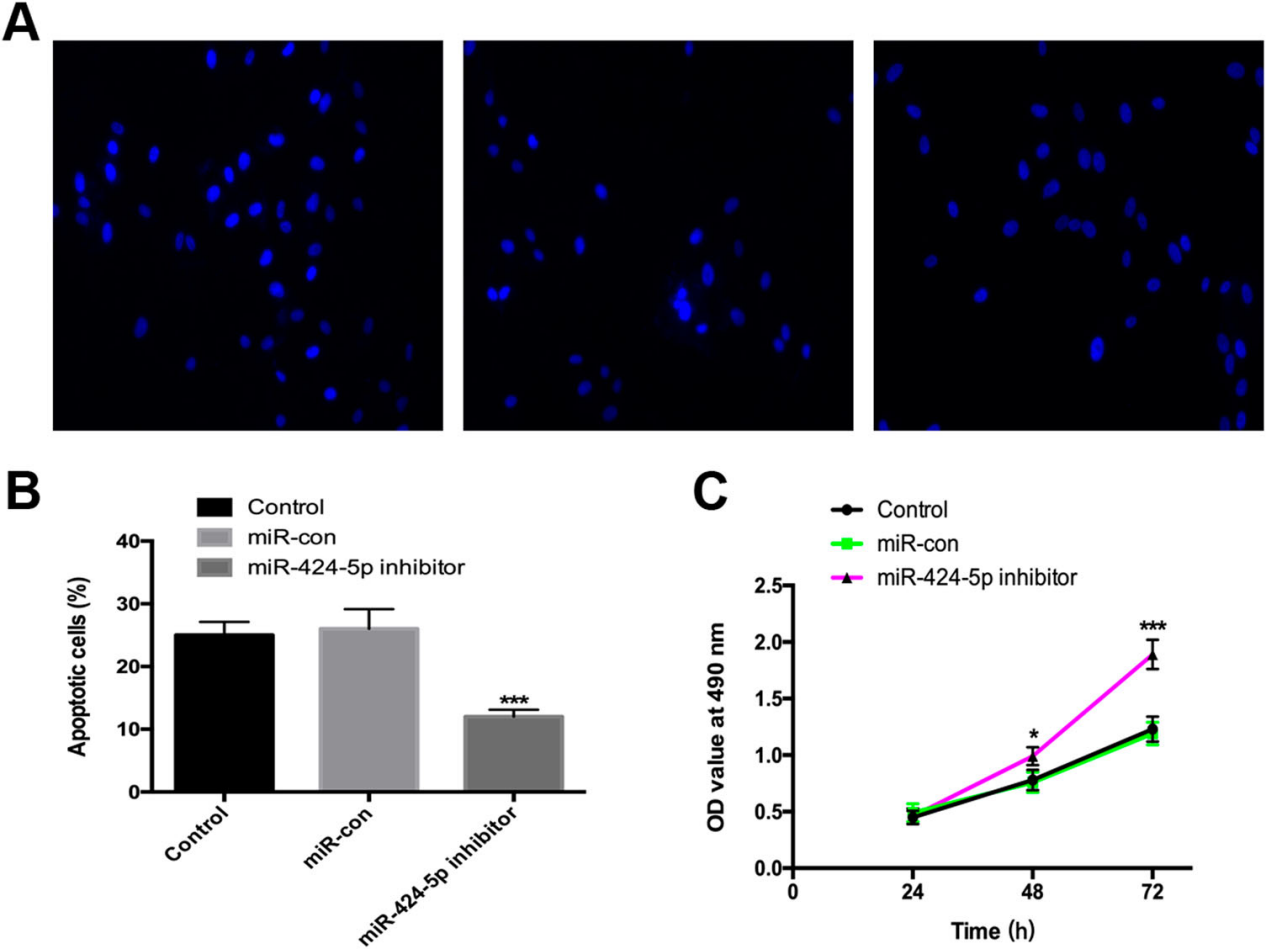
Inhibition of miR-424-5p promotes cell viability and suppresses cell apoptosis in degenerative NP cells. (A and B) A Hoechst staining kit was used to detect apoptotic cells following miR-424-5p silencing. (C) Cell viability was detected by using MTT assays. Experiments were performed three times. *, *p* < 0.05; ***, *p* < 0.001.

We used a Hoechst staining kit to detect the level of apoptosis in different groups, and the results showed that inhibition of miR-424-5p decreased the level of apoptosis (Figure 3A and B). MTT assays were applied to detect the viability of degenerative NP cells. Suppression of miR-424-5p remarkably enhanced cell viability in degenerative NP cells (Figure 3C).

### Inhibition of mir-424-5p decreases the levels of cleaved caspase-3 and cleaved caspase-9 in degenerative NP cells

**Figure 4.**
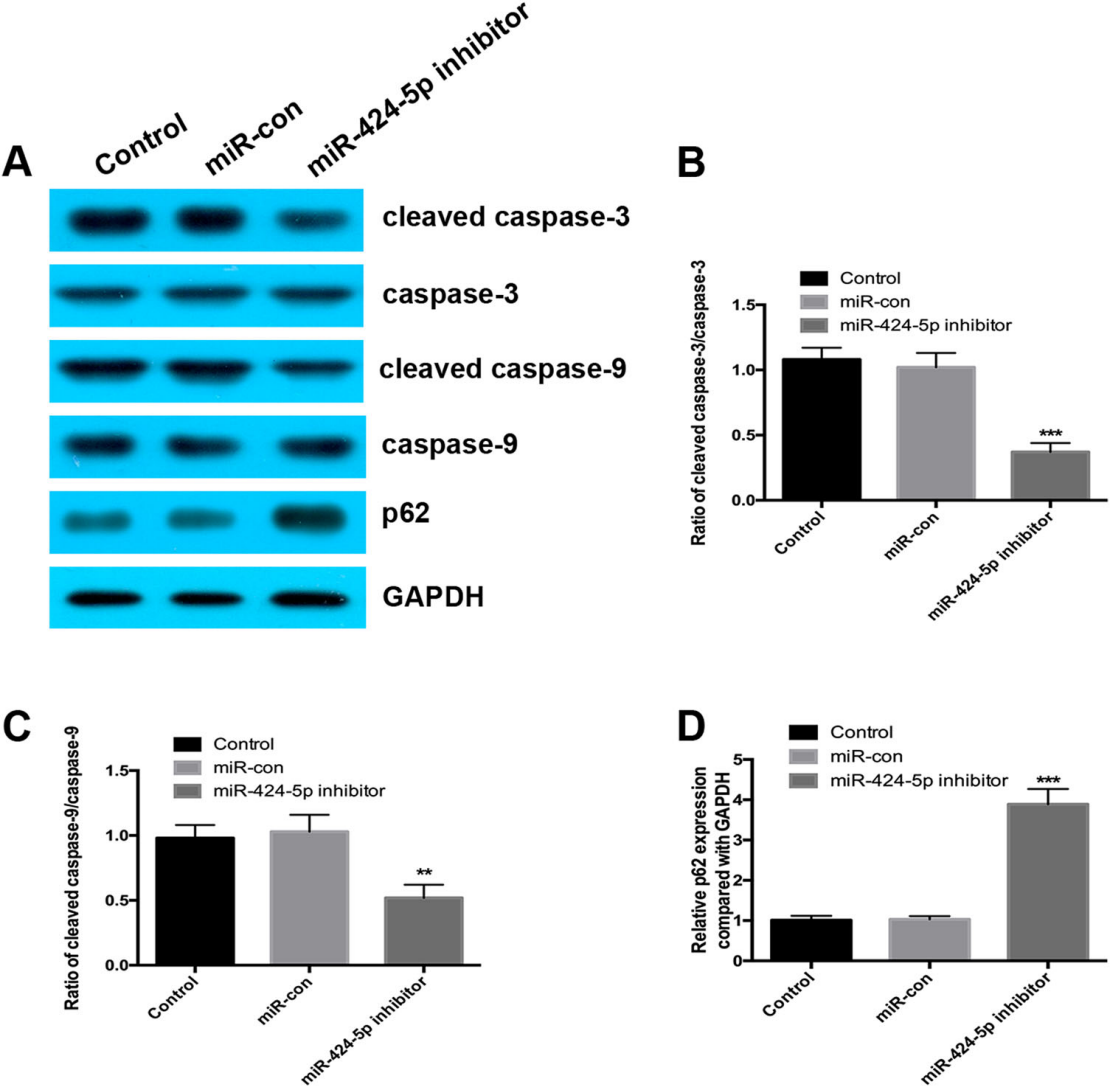
Inhibition of miR-424-5p decreases the levels of cleaved caspase-3 and cleaved caspase-9 in degenerative NP cells. (A-C) Western blotting was used to detect the protein expression of cleaved caspase-3, cleaved caspase-9, caspase-3, caspase-9 and p62 in NP cells. (D) Real-time PCR was used to detect the expression of p62 in NP cells. Experiments were performed three times. **, *p* < 0.01; ***, *p* < 0.001.

Our results further revealed that inhibition of miR-424-5p significantly attenuated the levels of cleaved caspase-3 and cleaved caspase-9 in degenerative NP cells (Figure 4A-C). Importantly, the miR-424-5p inhibitor increased the p62 protein expression level (Figure 4D). These results indicated that miR-424-5p silencing reduced the level of apoptosis by inactivating the caspase-9/caspase-3 signalling pathway.

### miR-424-5p directly targets Bcl2

**Figure 5.**
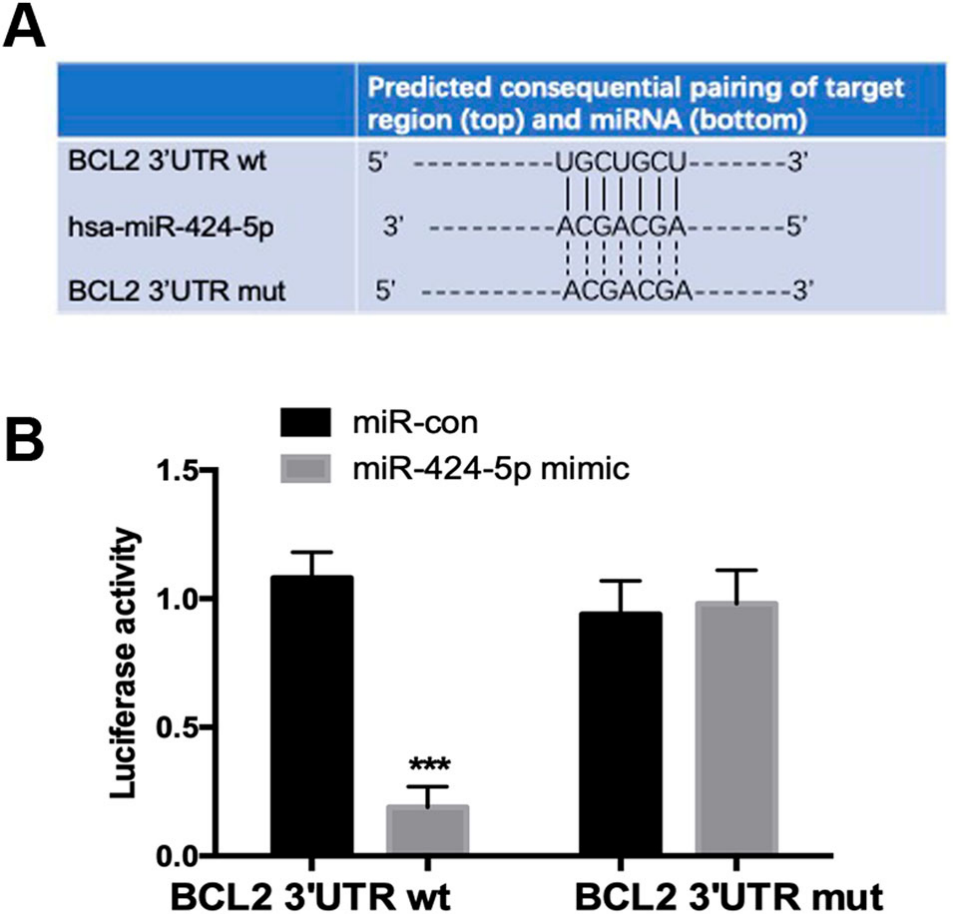
miR-424-5p directly targets Bcl2. (A) Predicted miR-424-5p target sequence in the 3’UTR of Bcl2. (B) A luciferase reporter assay was used to detect the binding between miR-424-5p and the 3’UTR of Bcl2. Experiments were performed three times. ***, *p* < 0.001.

Bcl2 is the predicted target of miR-424-5p, which was analysed by the TargetScan database (Figure 5A). The luciferase reporter assay showed that miR-424-5p could directly bind the WT 3’UTR of Bcl2, and the binding ability was significantly decreased when the binding sites were mutated (Figure 5B). All the results suggested that miR-424-5p directly targeted and regulated Bcl2.

## Discussion

An increasing number of studies have suggested that miRNAs play more important roles in the progression of IDD by regulating cell proliferation and apoptosis. For example, miR-573 is downregulated in degenerative NP cells, and its target gene Bax is upregulated; overexpression of miR-573 could enhance cell viability and inhibit apoptosis by targeting Bax in NP cells (Wang et al. 2019). Meta-analysis data showed that miR-574-3p, miR-199a-5p, miR-640 and miR-551a were frequently upregulated in IDD compared with that of control tissues, while miR-483 was frequently downregulated (Sherafatian et al. 2019). However, the roles of miR-424-5p in the progression of IDD have not been elucidated.

In our study, we found that the expression of miR-424-5p was increased in human IDD samples and that its target gene Bcl2 was decreased. Very importantly, the expression levels of miR-424-5p and Bcl2 were negatively correlated. Moreover, inhibition of miR-424-5p significantly upregulated Bcl2 expression at both the mRNA and protein levels. Furthermore, inhibition of miR-424-5p remarkably suppressed apoptosis and promoted cell viability in NP cells. A luciferase reporter assay confirmed that miR-424-5p could bind the 3’UTR of Bcl2. In summary, our findings suggested that miR-424-5p negatively regulated Bcl2.

Apoptosis, one type of programmed cell death, is stimulated by DNA damage and inflammation, and it is involved in the progression of IDD (Vo et al. 2016; Zhang et al. 2016). Bcl2 is an apoptosis suppressor that regulates mitochondrial membrane permeability and downstream caspases (Llambi and Green 2011). Recent studies revealed the roles of Bcl2 in the progression of IDD. LncRNA GAS5 overexpression in NP cells led to an increase in caspase-3 and a decrease in Bcl2 and further promoted the apoptosis of NP cells (Wang et al. 2019). Silencing TREM2 could inhibit NP cell apoptosis by reducing Bax and upregulating Bcl2 (Bai et al. 2018). Bcl2 has been shown to be targeted and regulated by miR-143 in human NP cells (Zhao et al. 2017), and in the present study, we found that miR-424-5p could also target Bcl2 and regulate the apoptosis of NP cells.

## Conclusion

Our study indicated that miR-424-5p was upregulated and Bcl2 was downregulated in IDD samples and that miR-424-5p silencing inhibited apoptosis and promoted cell viability of NP cells by targeting Bcl2. Our findings showed that miR-424-5p might be a novel therapeutic target for IDD.
